# Diseases of the salivary glands in infants and adolescents

**DOI:** 10.1186/1746-160X-6-1

**Published:** 2010-02-15

**Authors:** Maik Ellies, Rainer Laskawi

**Affiliations:** 1 Department of Otorhinolaryngology, Head and Neck Surgery, University of Göttingen, Göttingen, Germany

## Abstract

**Background:**

Diseases of the salivary glands are rare in infants and children (with the exception of diseases such as parotitis epidemica and cytomegaly) and the therapeutic regimen differs from that in adults. It is therefore all the more important to gain exact and extensive insight into general and special aspects of pathological changes of the salivary glands in these age groups. Etiology and pathogenesis of these entities is still not yet fully known for the age group in question so that general rules for treatment, based on clinical experience, cannot be given, particularly in view of the small number of cases of the different diseases. Swellings of the salivary glands may be caused by acute and chronic inflammatory processes, by autoimmune diseases, by duct translocation due to sialolithiasis, and by tumors of varying dignity. Clinical examination and diagnosis has also to differentiate between salivary gland cysts and inflammation or tumors.

**Conclusion:**

Salivary gland diseases are rare in childhood and adolescence. Their pattern of incidence differs very much from that of adults. Acute and chronic sialadenitis not responding to conservative treatment requires an appropriate surgical approach. The rareness of salivary gland tumors is particularly true for the malignant parotid tumors which are more frequent in juvenile patients, a fact that has to be considered in diagnosis and therapy.

## Introduction

Diseases of the salivary glands are rare in infants and children (with the exception of diseases such as parotitis epidemica and cytomegaly) and the therapeutic regimen differs from that in adults. It is therefore all the more important to gain exact and extensive insight into general and special aspects of pathological changes of the salivary glands in these age groups. Previous studies [1-3] have dealt with the clinical distribution pattern of the various pathological entities in infants and older children.

According to these studies, important pathologies in these age groups are acute and chronic sialadenitis (with special regard to chronic recurrent parotitis) and secondary inflammation associated with sialolithiasis [2,4-6]. The etiology and pathogenesis of these entities in young patients, however, are still not yet sufficiently understood, so that therapeutic strategies based on extensive clinical experience cannot be defined, particularly in view of the small number of patients in the relevant age groups. The acute forms of sialadenitis are mainly caused by viral or bacterial infections. The predominant cause of parotid swelling in infancy is parotitis epidemica [7]. This disease has its peak incidence between the ages of 2 and 14 [8]. Acute inflammation of the parotid gland, with evidence of Staphylococcus aureus, is often seen in neonates and in children with an underlying systemic disease accompanied by fever, dehydration, immunosuppression and general morbidity [4,9]. Acute inflammation of the submandibular gland, as opposed to that of the parotid is usually due to a congenital anomaly of a salivary duct or an excretory duct obstruction [4,10]. Reports on sialolithiasis in infants and adolescents, however, are very scarce and are mostly presented as rarities in clinical case reports [6]. For chronic sialadenitis the predominant etiological factors are secretion disorders and immunological reactions [11]. The pathogenesis of chronic recurrent parotitis has still not been completely elucidated and is, next to mumps, the most frequent sialadenitis in infancy [12].

Neoplastic changes are very rare in children and adolescents, compared to salivary gland inflammations [1]. Their annual incidence in all juvenile age groups is 1 to 2 tumor cases in 100,000 persons. According to Eneroth [13] salivary gland tumors make up 0.3% of all human tumors, and less than 10% of all juvenile head and neck tumors are located in the salivary glands [14]. Only 1% of all head and neck tumors originate in the salivary glands, regardless of patient age [15]. Not only makes this low incidence the establishment of a generally applicable therapeutic regime difficult; this task is not made easier by the circumstance that not more than 5% of all salivary gland tumors are found in the age group of up to 16 years [16]. As a consequence therapies very often lean on experience gained in the last decades from long-term studies for the treatment of adult patients.

Primary dysgenetic, and secondary, acquired salivary gland cysts, and other malformations of the salivary glands have to be distinguished early and without doubt from specific benign and, above all, malignant lesions by pathohistological examination [17].

### Inflammatory Diseases of the salivary glands

Inflammatory salivary gland diseases, next to benign neoplasms, are the most frequent causes of salivary gland swelling in juvenile age [3]. The acute forms of sialadenitis are bacterial and viral in origin. In childhood, the parotid gland is most frequently affected by acute bacterial inflammation [2,4]. Predominant among the bacterial pathogens are group A streptococci and Staphylococcus aureus. This is supported by our own findings [18]. Typical viral diseases are parotitis epidemica and cytomegaly. These clinical entities, because they are well-known, were not included in our review.

#### Sialadenitis and sialolithiasis

In the extensive study of Zenk et al. [19] on 635 patients, sialolithiasis of the submandibular gland was most frequent in patients between 31 and 55 years of age, and only 6.1% of all patients with sialolithiasis of the cephalic salivary glands were younger than 20 years. Judging from the reports in the literature, sialolithiasis is rarely observed in infants and adolescents [20,21]. In a review covering a period of approximately 100 years [22], there were only 21 documented cases of sialolithiasis of the submandibular gland in children between 3 weeks and 15 years of age. As in adults, the leading symptom is a painful swelling of the afflicted gland that abates postprandial. The literature describes sialolithiasis in infants mostly in single case reports. The youngest documented case in a single case report is that of a two-year-old child [23]. Walsh and Robson [6] reported the spontaneous passage of a submandibular salivary duct calculus in a 9-year-old girl.

The children documented in our own study [18] constitute a selected group of patients, since they presented at our clinic for operative therapy in well-defined cases. Concrements were located intraglandularly or found in the proximal portion of the Wharton's duct (submandibular duct) in 66.7% of cases, and in 33.3% they were localized outside the gland in the vicinity of the hilus and of the distal portion of the excretory duct. Following submandibulectomy or slitting of the Wharton's duct the patients were permanently free of symptoms. In this context, Zenk et al. [24] describe a technique with slitting of the Wharton's duct in its entirety, identification and preservation of the lingual nerve and enoral stone removal. Concrements located within the gland are dealt with by excision of the submandibular gland.

We recommend removing sialoliths from the distal portion of Wharton's duct by slitting under perioperative antibiotic cover. This procedure is also the first choice in enoral proximally palpable stones, if the course of the lingual nerve is taken into account. Our therapeutic concept which is adapted to the location of the stone has yielded satisfactory postoperative results and proved to be effective in the treatment of sialolithiasis of the submandibular gland in childhood and adolescence.

We observed only one instance of sialolithiasis of the parotid gland in a 4-year-old boy (2.2%) [18]. Zenk et al. [24] found seven cases (1.1%) among their patients less than 25 years. The same authors found amongst the total of 635 patients studied, a solitary parotid gland stone in a 4-year-old girl and a 2-year-old boy. Due to the rarity of cases in this age group the therapy of parotid sialolithiasis has to be adapted from that employed for adults, in close cooperation with pediatric colleagues. Acute sialadenitis of the submandibular gland without evidence of concrement can be managed by temporary drainage under antibiotic cover, similar to the therapy of acute parotitis. In our patients, this was done in the course of therapy for parotid abscess.

#### Chronic recurrent parotitis

Chronic recurrent parotitis is, next to mumps, the most common inflammatory salivary gland disease in childhood and adolescence [8,11,12,25]. After sialolithiasis of the submandibular gland, the group of patients with chronic recurrent parotitis was the second largest in our study. According to Grevers [26] this disease has a juvenile and an adult course of progression. Its pathogenesis is not fully elucidated. There are conflicting opinions in the literature as to a possible connection with congenital [9,27], acquired or multifactorial inflammation-induced stenosis and ectasia of the duct system [26], congenital duct anomalies [27,28], and post-infectious factors [19]. In addition, the involvement of autoimmune processes has been suggested [29]. Chronic sialectatic parotitis (CSP) in infants and adolescents is a special entity [11] whose pathogenesis may be associated with immunopathological reactions of MALT (mucosa membrane associated lymphoid tissue). This would support the hypothesis [30] of an autoimmune etiology. According to Galili and Marmary [12] the disease starts between the third and sixth year of life. Accordingly, our analysis shows a peak incidence in the group of 5- to 10-year-old patients.

As mentioned above, the patients admitted to our clinic in the period covered by our own studies [18] were mainly those with frequent and extremely prolonged episodes of chronic recurrent parotitis with an indication for surgery. Between 1966 and 2000, a number of invasive and surgical therapeutic concepts have been applied in the treatment of this disease, internationally as well as in our department at the University of Göttingen. Total parotidectomy was performed in 54.5% of the cases and was without long-term complications. In two patients, the disease had healed spontaneously by the onset of puberty. Instillation of a fibrin-glue/gentamycin mixture into the Stenon's duct was eventually found to be unsuitable [31], and long-term results after tympanic neurectomy were unsatisfactory [28]. Consequently, both procedures were abandoned.

Based on our present knowledge, we recommend symptomatic measures combined with the administration of antibiotics and analgesics for the initial treatment of juvenile chronic recurrent parotitis. Sialendoscopical removal of inspissated proteins in the stenon's duct can be helpful, too. We feel justified in making this recommendation in view of this disease's tendency to spontaneous healing before puberty [3]. Follow-up and control examinations in short intervals are desirable in all patients with successful initial conservative treatment to detect early signs of recurrent parotitis by clinical and ultrasound examinations. We stress the importance of total parotidectomy when inflammatory episodes recur frequently (with certain restrictions in prepubertal patients here as well) as the only expedient option in cases of drug resistance. All of our surgically treated patients have remained free of complaints. The literature reports lasting success rates of 80 to 100% [32,33]. Prior to the operation, the parents must be thoroughly informed about the purpose and technique of the procedure, possibly also in the presence of the child. Specific mention must be made of the risk of temporary facial paresis and of the development of Frey's syndrome.

Among our patients with chronic recurrent parotitis we had two instances of temporary facial paresis following parotidectomy [18]. This was already receding before the patients were discharged from the hospital and was no longer visible three months after surgery. This complication is particularly not uncommon in patients with frequent inflammatory episodes and consecutive fusion between parenchyma and nerve fibers. We saw no instance of persisting postoperative nerve injury or symptomatic Frey's syndrome following total extirpation of the parotid gland due to chronic sialadenitis.

### Tumors of the salivary glands

Due to the fact that tumors of the salivary glands in childhood and adolescence are a rare disease, it is in our opinion not very easy to make a comparison with a similar adult population. On the one hand, it is not possible to get significant information especially due to the high variety of different tumors. On the other hand, the problem of a retrospective clinical investigation is sometimes a lack of specific information, which makes it hard to determine a really similar adult population.

Lesions of the major cephalic salivary glands, with the exception of mumps and cytomegaly, are unusual in children and adolescents and may give rise to a number of different tentative diagnoses. Since malignant salivary gland tumors are relatively more frequent in young person's than in adults, a safe diagnosis has to be made quickly and without delay. This is even more important as according to Ussmüller et al. [34] about one half of all juvenile salivary gland tumors may be malignant tumors.

#### Benign Neoplasms

According to a study by Luna et al. [35] on tumor incidence in the salivary glands, based on data of 6 centers comprising 9823 patients, 3.3% of all neoplasms, regardless of their dignity, are found in persons younger than 16 years. Castro et al.[36] found among 2135 cases 38 young patients between 5 and 16 years with salivary gland tumors, corresponding to an incidence of only 1.8%.

**Figure 1:**
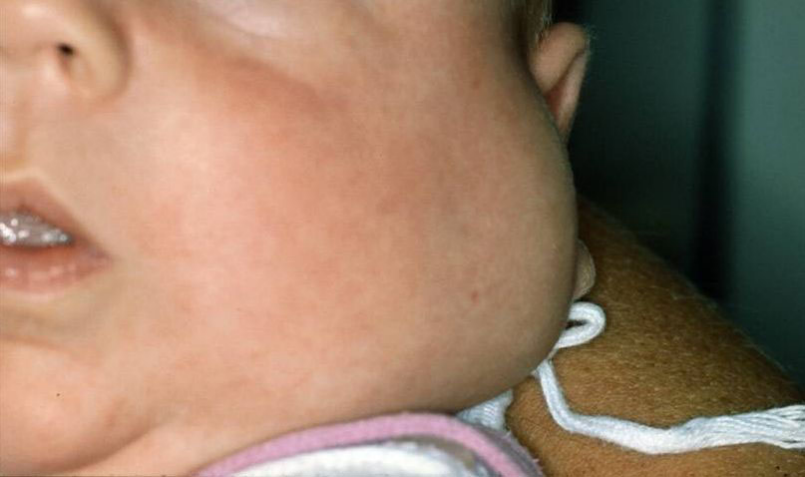
**Infant shows a haemangioma on the left side of the neck**.

Due to our own studies [37] there were 40 patients with benign lesions, 79% of which were localized in the parotid gland, with a predominance of pleomorphic adenomas (60%) in the age range investigated. This is in accordance with a number of other reports [14,38]. Luna et al. [35], too, state that pleomorphic adenomas are the most frequent benign epithelial tumors in childhood. Other teams, however, saw a majority of non-epithelial neoplasms, haemangioma and lymphangioma (Fig. 1), in the group of benign growths [39]. In a study of 782 cases examined with respect to histological classification, Ussmüller et al. [34] found a dominance of non-epithelial tumors in the first years of life. In still another study [40] the non-epithelial tumors were the most frequent benign neoplasms in the parotid region (50%) in newborns and infants. This agrees well with our findings. We saw 66.6% of non-epithelial tumors (haemangioma, haemangiolymphoma) in infants. Eneroth und Hjertman [41] found 75-85% of all benign lesions in the parotid, and 10% in the submandibular gland. This is very similar to our observations (parotid gland: 92.5%, submandibular gland: 7.5%) [37].

The rarity of salivary gland tumors in young people makes it impossible for just one ENT department to gain solid experience in their diagnosis and therapy. It should be the aim of reports on therapeutic experience to present treatment strategies and provide the international otolaryngological scene with operation results. Our particular interest was focused on pleomorphic adenoma, due to its high incidence clinically the most important tumor for the development of surgical approaches. In the early years covered by our report, prior to introduction of lateral, respectively total, parotidectomy, always with preparation of the parotid plexus, we saw tumor recurrences in 80% of cases following enucleation alone. This result resembles that of another study [42] which reported an incidence of 20-45% of recurrences after enucleation. According to Leverstein et al.[43] most recurrences arose from inadequate operation techniques. Arnold [44] has nicknamed pleomorphic adenoma as a "wolf in sheep's skin": Enucleation carries the risk of tumor cell transfer, respectively incomplete tumor removal, since a large percentage of pleomorphic adenomas are not completely encapsulated or are enveloped only by a thin layer of connective tissue.

The relatively high proportion of recurrences which we observed despite correct operation techniques may be explained by the fact that the majority of patients presented at our clinic for second operations after primary surgery elsewhere. After introduction of operation microscope-controlled techniques and after performance of lateral parotidectomy for laterally localized adenomas the frequency of recurrences was dramatically reduced to 2% [45]. When using operation microscope-based techniques at our clinic, recurrences were virtually absent after primary operations. We therefore recommend the following procedure for surgery of parotid pleomorphic adenoma.

The therapy of pleomorphic adenoma consists of lateral parotidectomy with en-bloc excision of the tumor within the surrounding tissues, preserving facial nerve integrity. This is the smallest operation and helps to minimize the risk of recurrences [46]. The important first preoperative diagnostic step in young patients is sonographic examination of the parotid region. Fine-needle aspiration biopsy, routinely used in adults for differential diagnosis, is also applicable in children, and a safe decision for further therapy is in most cases also possible. For deep-lying tumors, total parotidectomy with preservation of the facial nerve is the therapy of choice. The majority of pleomorphic adenomas is localized in the lateral portion of the parotid gland [43,45]. McGurk et al. [45] found even 90% of all adenomas in the superficial parotid lobe, situated laterally of the parotid plexus. In our retrospective study of operation reports, however, we found a higher proportion of tumors (47.6%) in the deep lobe of the parotid gland, medially of the parotid plexus. The superficial part of the parotid harbored 42.9% of all pleomorphic adenomas [37].

#### Malignant Neoplasms

**Figure 2:**
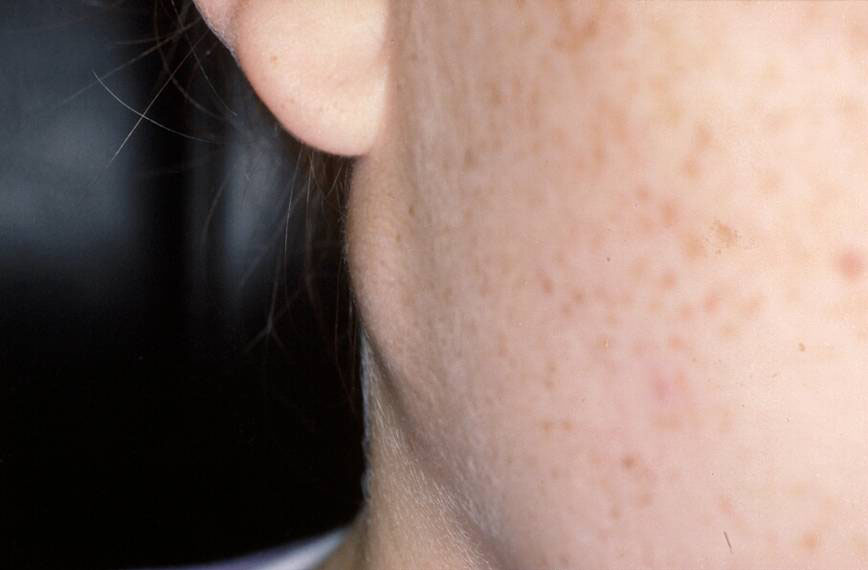
**Mucoepidermoid carcinoma of the right parotid gland occurred in a young girl**.

Although malignant salivary gland tumors are uncommon in children and adolescents, clinical diagnosis has to be made very carefully, since compared with adults the proportion of malignancies among all neoplasms is relatively high. In childhood 80-90% of all malignant lesions of the salivary glands are made up by mucoepidermoid carcinomas (Fig 2), adenoid-cystic carcinomas and acinic cell carcinomas. The corresponding figure in adults is only 45%. While Eneroth [47], in his study of incidence and prognosis of 2632 patients with tumors of the major and minor salivary glands, found an incidence of 15-25% of malignant neoplasms for adults, many teams reported a significantly higher relative proportion in young patients. In the age range studied by us, 50% of all salivary gland tumors are malignant if haemangiomas and lymphangiomas are not included [36,48]. Schuller and McCabe [49] report a slighly higher incidence of 57.1%.

In adults, 65-75% of the epithelial neoplasms are benign in nature, but in children only between 50 and 60% [48,49]. Many publications agree that mucoepidermoid carcinoma is the most abundant malignant salivary gland tumor in young patients [14,38]. This is confirmed by our own investigations [37]. Within the group of salivary gland malignancies we found 33.3% of mucoepidermoid carcinomas, followed in frequency by 25% each of adenoid-cystic carcinomas and embryonic rhabdomyosarcomas. The highest incidence of mucoepidermoid carcinoma is found in the second decade of life, while the tumor is rare in the first [50]. Determination of histological subtypes yielded 3 low-grade (highly differentiated) mucoepidermoid carcinomas (75%) that have a high 5-year survival rate (more than 95%) according to Chomette et al. [51]. This favorable prognosis was confirmed by the results of follow-up of patients with this tumor who were treated at our clinic. One patient died in the postoperative observation period from a high-grade (low differentiation) mucoepidermoid carcinoma.

The characteristic and determining factor in the group of adenoid-cystic carcinomas is their perivascular and perineural tendency for infiltration [52] which makes prognosis less favorable with a 5-year survival rate of 60% and a 10-year survival rate of 40% in all age groups [53]. During a follow-up period of 25 years we did not lose a single patient operated for this tumor.

In their survey of patients younger than 20 years, Byers et al. [54] measured a 5-year survival rate of 50% for patients with acinic cell carcinoma, including high-grade carcinomas in the statistical evaluation. Data from our clinic on this malignancy include only patients with low-grade acinic cell carcinoma who had a 5-year survival rate of 100%.

Embryonic rhabdomyosarcomas of the cephalic salivary glands are rare [55] and have a poor prognosis since the patients present in most cases with already far advanced tumor invasion. Rogers et al. [56] were reporting on 9 patients between 1 and 13 years, 77.7% of whom died 6 to 9 months after diagnosis. This is in accordance with our experience with young patients with an embryonic rhabdomyosarcoma of the parotid gland. All 3 patients died within a few months after the initial diagnosis. In the extended study of Castro et al. [36] the 5- and 10-year survival rates for salivary malignancies, with the exception of sarcomas, were 94, respectively 95%.

Our surgical concept and our favorable long-term results show that total or radical parotidectomy, sometimes including extended resection of neighbouring structures, is the best therapy for malignant parotid tumors in children, with relatively few complications throughout the follow-up period [37]. No statement can be given about the outcome of radiation therapy because none of the patients in our investigation received it. However, for the establishment of an individual concept of oncological therapy (parotidectomy, neck dissection, chemotherapy, radiotherapy), interdisciplinary cooperation with the pediatrician is mandatory.

## Conclusion

Salivary gland diseases are rare in infants and children. Acute and chronic sialadenitis not amenable to conservative therapy requires surgical treatment. The clinical course of chronic recurrent sialadenitis in children has a great potential for spontaneous healing, but in a number of cases it does not permit waiting for spontaneous healing until puberty but requires surgical intervention. As these diseases are rarer in young people than in adults, it is difficult to establish universally valid therapeutic guidelines. Salivary gland tumors, rare in childhood and adolescence, differ in their incidence and dignity between juvenile and adult patients. This is particularly true of parotid malignancies which are more frequent in young persons. This fact has to be taken into account in diagnosis and therapy. Long-term multicenter studies for comparison of treatment strategies are needed in the coming decades to guarantee further optimization of tumor management on a profound clinical and scientific basis, for the benefit of our young patients.

## Abbreviations

CSP: chronic sialectatic parotitis; MALT: mucosa membrane associated lymphoid tissue.

## Consent

It is stated that informed written consent was obtained for publication of the patients images.

## Competing interests

The authors declare that they have no competing interests.

## Authors' contributions

The authors issued the whole manuscript. Both authors have read and approved the final manuscript.
